# Fewer clouds in the Mediterranean: consistency of observations and climate simulations

**DOI:** 10.1038/srep41475

**Published:** 2017-02-02

**Authors:** Arturo Sanchez-Lorenzo, Aaron Enriquez-Alonso, Josep Calbó, Josep-Abel González, Martin Wild, Doris Folini, Joel R. Norris, Sergio M. Vicente-Serrano

**Affiliations:** 1 Instituto Pirenaico de Ecología, Consejo Superior de Investigaciones Científicas (IPE–CSIC), Zaragoza, Spain; 2 Department of Physics, University of Girona, Girona, Spain; 3 Institute for Atmospheric and Climate Sciences, ETH Zürich, Zürich, Switzerland; 4 Scripps Institution of Oceanography, University of California, San Diego, La Jolla, CA, USA

## Abstract

Clouds play a major role in the climate system, but large uncertainties remain about their decadal variations. Here we report a widespread decrease in cloud cover since the 1970 s over the Mediterranean region, in particular during the 1970 s–1980 s, especially in the central and eastern areas and during springtime. Confidence in these findings is high due to the good agreement between the interannual variations of cloud cover provided by surface observations and several satellite-derived and reanalysis products, although some discrepancies exist in their trends. Climate model simulations of the historical experiment from the Coupled Model Intercomparison Project Phase 5 (CMIP5) also exhibit a decrease in cloud cover over the Mediterranean since the 1970 s, in agreement with surface observations, although the rate of decrease is slightly lower. The observed northward expansion of the Hadley cell is discussed as a possible cause of detected trends.

Clouds affect the global energy balance as they reflect a large fraction of the Sun’s incoming radiation and at the same time absorb and emit longwave radiation^1^,^2^. Despite their relevance, large uncertainties remain with respect to the response and feedbacks of the clouds to anthropogenic forcing. Consequently they are considered as one of the main sources of uncertainty for climate sensitivity and future climate scenarios^3^,^4^,^5^.

In addition, there is a lack of knowledge regarding the decadal variations and trends of clouds^6^. Surface visual observations from human observers provide long-term data since the mid-19^th^ century, but these records are available only at select meteorological stations, especially over land areas^7^. Equally, these records are somewhat subjective and their time series are often inhomogeneous, which can jeopardize the obtained results^8^,^9^,^10^. Nevertheless, a large number of studies have used these series in order to estimate trends of cloudiness on different spatial scales^7^,^11^,^12^,^13^,^14^,^15^.

Contrastingly, satellite products can provide records of cloud changes on a global scale, but the study of trends is only possible since the 1980 s^16^. A lack of homogeneity of the records obtained from satellite-derived datasets has also been pointed out by different studies. This issue is usually attributed to changes in satellite zenith angle, problems with calibration of instruments, and changes in satellites themselves^17^,^18^,^19^. Recently, a statistical method has been applied to remove spurious trends in the total cloud cover (TCC) field of both the International Satellite Cloud Climatology Project (ISCCP) and the Pathfinder Atmospheres–Extended (PATMOS-x) datasets, which after the correction show a more realistic spatial pattern in their trends^20^.

The Mediterranean region is considered a “hot spot” in the context of climate change^21^, and projections show that strong warming and decreased precipitation is expected during the 21^st^ century^22^,^23^. There are a few regional studies of cloud trends in the Mediterranean area, and most of them have shown a tendency towards decreasing TCC during recent decades^24^,^25^,^26^, although increases have also been reported at some specific locations^27^,^28^. Future projections of TCC indicate that the decrease will continue during the 21^st^ century over the Mediterranean^29^,^30^, which would accompany the projected increase in surface downward solar radiation^29^ and the decrease in precipitation^31^.

Overall, a comprehensive analysis of the trends of TCC over the whole Mediterranean region is lacking, especially regarding seasonal differences. Equally, comparison of the trends derived from surface observations against other global gridded datasets such as satellite-derived and reanalyses products are also lacking. Finally, despite the interest of enhancing our confidence in future projections of TCC over the Mediterranean, the study of the ability of the climate models to reproduce the trends during the last decades has not been addressed yet. All these issues are addressed in this study with special emphasis on the following questions: Do observational data show a decrease in TCC on both annual and seasonal basis over the Mediterranean since the 1970 s? How widespread are these trends? Are the trends derived from satellite and reanalyses products in line with surface observations? How consistent are observations and simulations of TCC trends? For more details about the databases, see Data and Methods section.

## Decreasing cloud cover over the Mediterranean

**Figure 1:**
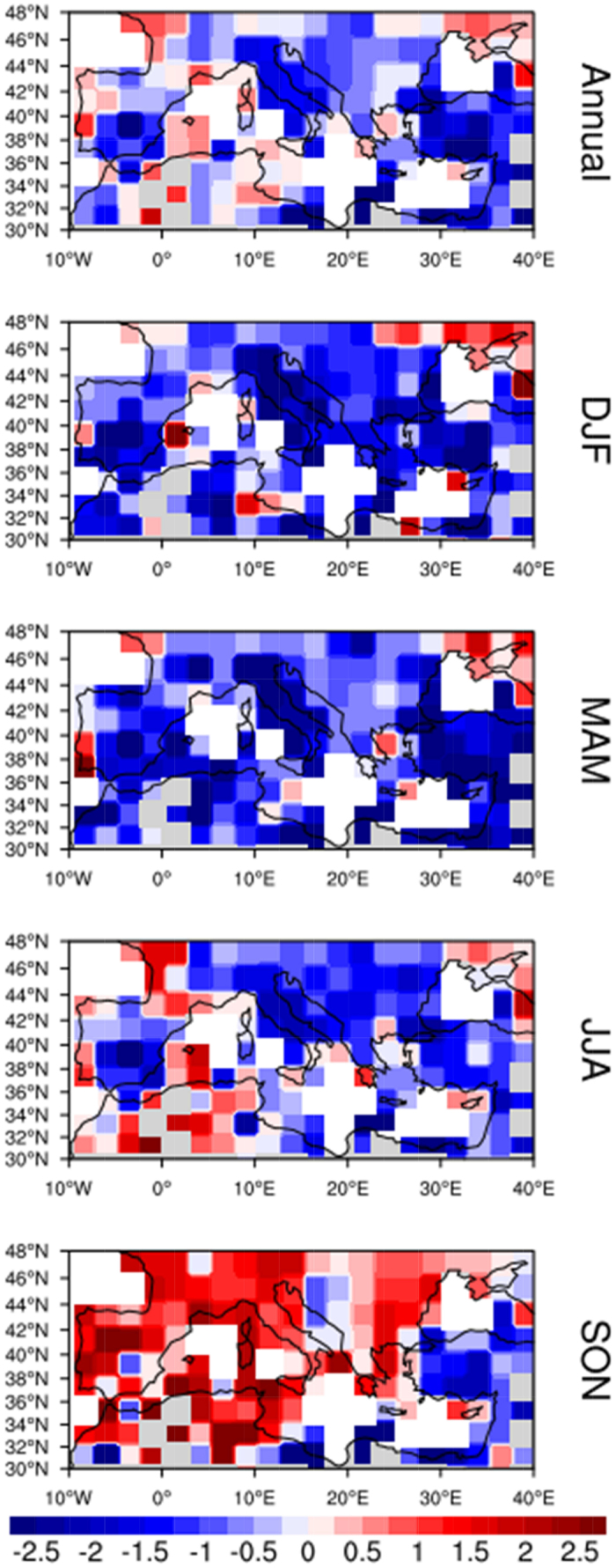
Linear trends of TCC, in each of the 2° × 2.5° grid cells, for the period 1971–2005 based upon the surface land observations given by EECRA. Units are % per decade. White cells correspond to areas with no data. The maps were created using the NCAR Command Language (Version 6.0.0) [Software] (2011). Boulder, Colorado: UCAR/NCAR/CISL/TDD. http://dx.doi.org/10.5065/D6WD3XH5.

Figure 1 shows the linear trends of ground-based (EECRA) observations of TCC for each cell over the Mediterranean region on annual and seasonal basis, for the 1971–2005 period. Annual trends are negative (blue) in general, although some small areas have positive (red) values. These negative annual trends were already reported in a previous study on a global scale^32^, although with much coarser resolution (10° × 10°). Interestingly, the negative trends are stronger and more widespread across the Mediterranean region during winter and spring. In these seasons, positive trends are restricted to northeast of the Black Sea, to southern Portugal, and to the western French coast. In summer, the pattern is less clear, with mostly decreasing trends in the East and some areas with positive trends in the West (including an area in North Africa). In autumn, the overall trend is positive, except for some areas (northeastern Africa, Anatolian Peninsula) with persistent negative trends.

**Figure 2:**
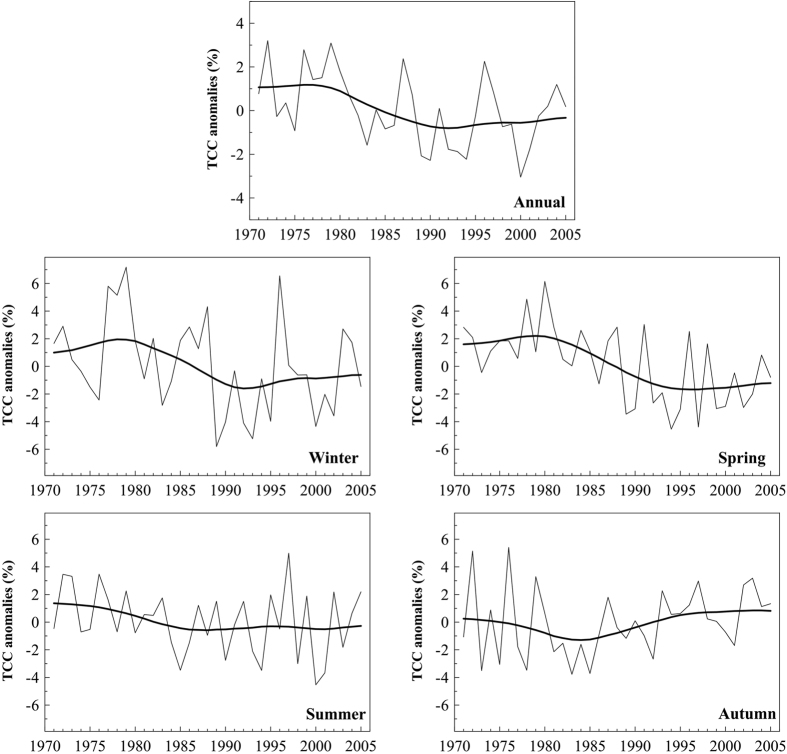
Time evolution of TCC anomalies in the Mediterranean region from 1971 to 2005 based on EECRA. Annual (or seasonal) values are shown by the thin line. The series are expressed as anomalies from the 1971–2000 mean: the anomaly is defined for each cell and month as the difference between the monthly TCC value and the mean value of TCC for that month and cell during the 1971–2000 period. The anomalies for the whole region are computed as the mean of those for all cells. The thick line is the smoothed evolution by applying a 13-year Gaussian low-pass filter.

The temporal evolution of the mean TCC over the whole region is presented in Fig. 2, which shows the mean annual (and seasonal) TCC anomaly series. The evolution of the annual anomalies clearly shows that the decreasing trend for the whole period (−0.63% decade^−1^, *p* < 0.05) is not monotonic, but instead is due to a substantial change in TCC between the late 1970 s and the early 1990 s (1971–1990: −1.3% decade^−1^, *p* < 0.05). Indeed, during the last fifteen years of the period selected (1990–2005) the mean TCC over the Mediterranean has remained essentially unchanged. On a seasonal basis (and for the complete 1971–2005 period), all series except autumn show similar decadal variations as the annual series, although the decrease is only statistically significant in spring (−1.42% decade^−1^, *p* < 0.01). Contrastingly, the autumn series shows a slight decrease from the 1970 s to the mid-1980 s, with an increase afterwards and a slight positive increase, albeit non-statistically significant, during the whole study period (+0.44% decade^−1^, *p* < 0.3).

On global land areas a mean TCC decrease of −0.4% decade^−1^ has been reported during the period 1971–2009^32^. The decreasing trend was especially remarkable in the tropics and mid-latitudes of both hemispheres, whereas a tendency towards increasing TCC was observed in equatorial and high-latitude areas. It is worth noting that the mean decrease on a global basis was, as in the Mediterranean region, mainly related to high positive anomalies during the 1970 s, with no relevant decadal variations afterwards.

In line with this global picture, many regional studies over mid-latitudes have shown a tendency towards negative trends of TCC for different regions since the mid-20^th^ century, such as for example in the US^33^, China^34^,^35^ and Poland^36^. In Australia, non-significant trends of TCC have been reported during the period 1957–2007^37^, although a decrease (−0.55% decade^−1^, yet non-significant) is observed if the study period is limited to the data after the 1970 s^32^,^37^.

## Can we trust satellite and reanalysis cloud products over the Mediterranean?

Trends for the reanalysis and satellite data series can be computed for a common period, which is 1984–2009, and compared with EECRA data, which are also available for this period. All these trends are presented in Figure S1, both on an annual and a seasonal basis and for the cells included in the EECRA mask. Some commonalities occur among the trends. In particular, all databases reveal negative TCC trends in spring across most of the Mediterranean region, while trends tend to be more positive in the Eastern Mediterranean area in autumn. Nevertheless, it is apparent that substantial differences also exist among the different databases. For example, ISCCP and CLARA tend to produce strong decreasing trends of TCC for most seasons.

**Figure 3:**
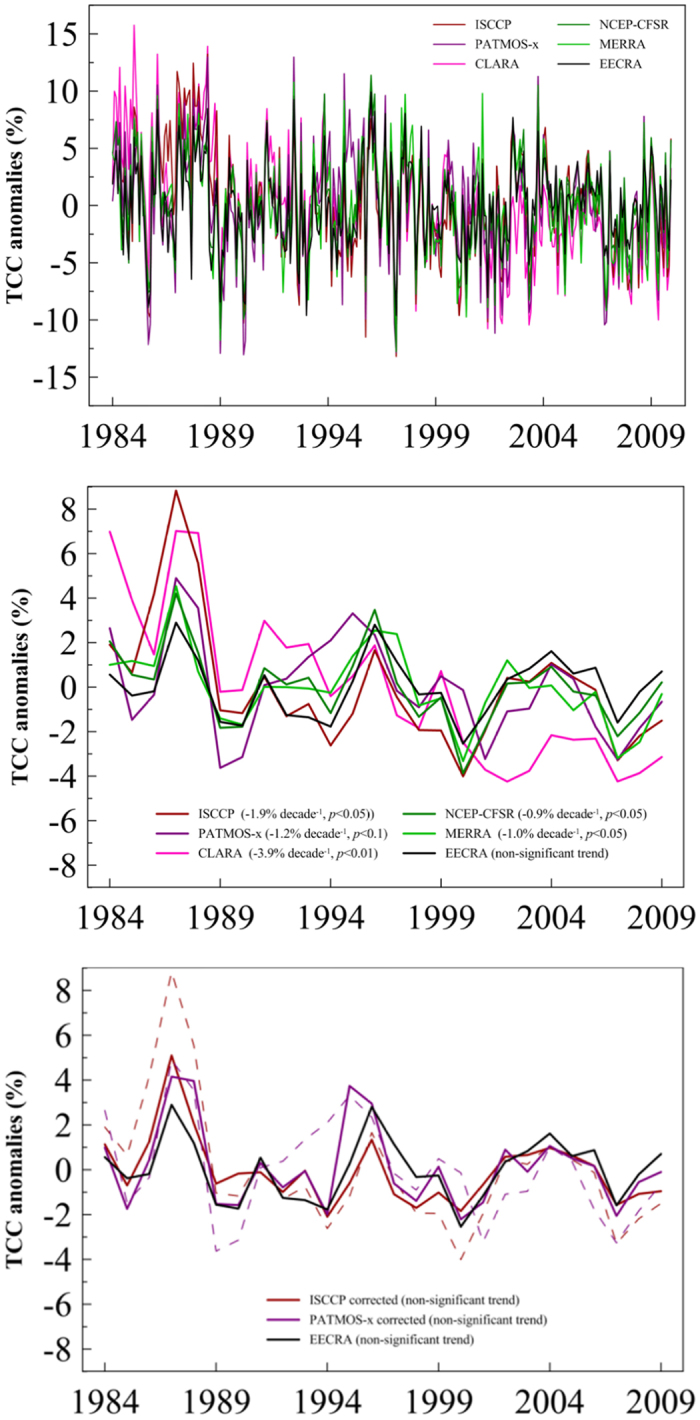
Evolution of mean monthly (top) and annual (middle) TCC anomaly series (period 1984–2009) over land in the Mediterranean region from ground-based observations (EECRA), satellite projects (ISCCP, CLARA, PATMOS-x), and reanalysis products (MERRA, NCEP-CFSR). (bottom) Evolution of mean TCC anomaly series for EECRA and corrected ISCCP and PATMOS-x records^20^, with dashed lines showing raw ISCCP and PATMOS-x data as in the middle panel. The series are expressed as anomalies from the 1984–2009 mean.

These general features can be seen in Fig. 3, where the month-to-month variability of the mean anomaly EECRA series for the whole Mediterranean region shows good agreement with the satellite and reanalysis products (Fig. 3, top), with correlation coefficients between EECRA and the other products ranging from 0.7 (CLARA) to 0.93 (NCEP-CFSR). Despite the similarities on interannual variations, the set of annual mean series (Fig. 3, middle) show disagreements in the trends, as all datasets give negative and statistically significant trends (≤ −1% decade^−1^), especially for ISCCP (−1.9% decade^−1^) and CLARA (−3.9% decade^–1^), whereas the EECRA trend is not statistically significant (*p* > 0.1) during the 1984–2009 period.

Even if all databases emphasize the decline of clouds in the Mediterranean during the last decades, the disagreement in rate and significance of the trends highlights the uncertainties of the different satellite and reanalysis products in capturing trends of clouds, in agreement with other recent studies over other regions^33^,^38^, even if the latter products can reproduce observed TCC trends in US^39^.

It is worth noting that ISCCP and PATMOS-x TCC products that have been corrected for artifacts^20^ show better correlations when compared against ECCRA data than the original data do (e.g., increasing from 0.71 to 0.72 and from 0.54 to 0.78 for ISCCP and PATMOS-x on an annual basis, respectively). Moreover, the corrected datasets have trends that are not statistically significant during the study period, in agreement with ground-based observations (Fig. 3, bottom). These results highlight the need of further research in order to improve satellite-derived and reanalysis products that currently lack high-quality records for reproducing decadal variations in cloudiness. Thus, it is desirable to validate these products by inter-comparing them^16^ and with traditional ground-based observations of clouds, which can help to distinguish between real climate variability and artifacts^33^,^39^.

## Can climate models reproduce this decreasing trend since the 1970s?

**Figure 4:**
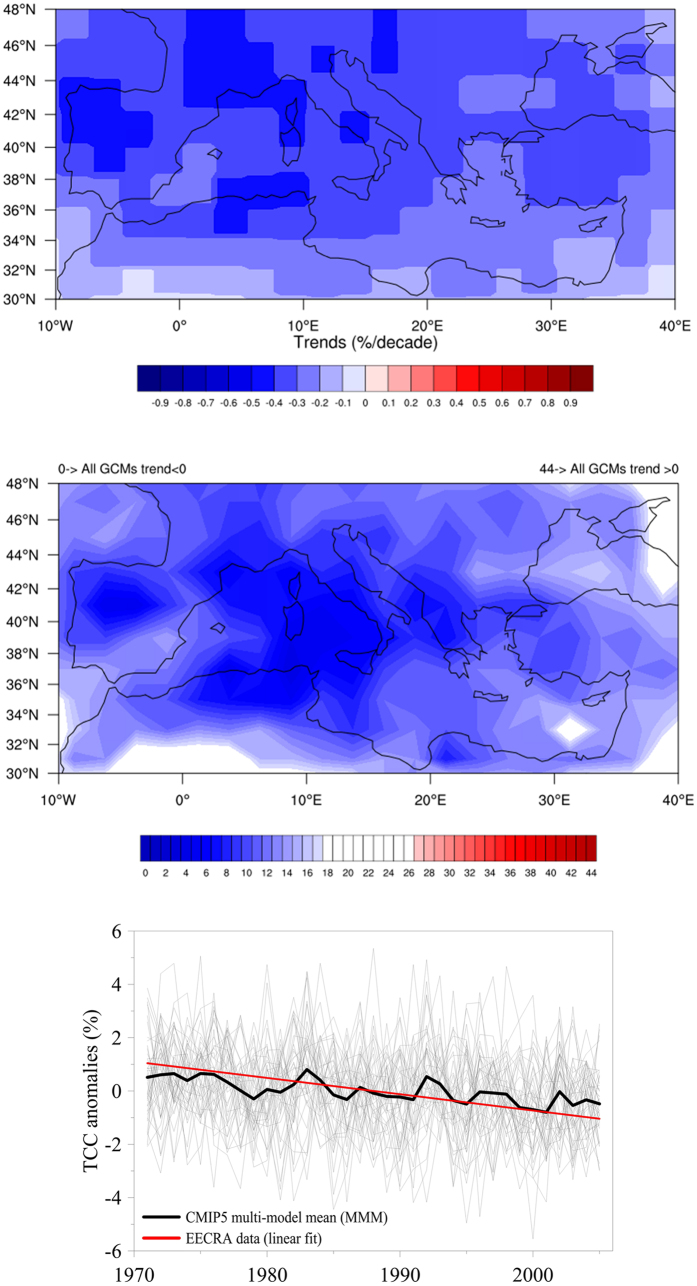
(top) Mean TCC trends for the period 1971–2005 obtained by averaging the trends of the 44 GCM considered. Note that the scale is different from that in Fig. 1 and S1. (middle) Number of GCMs that give a positive TCC trend; therefore, the bluer a cell is tinted, the more robust the negative trend given by the ensemble of the GCMs. (bottom) Time evolution of the annual TCC anomaly for the whole Mediterranean region (using the EECRA mask) during the 1971–2005 period for each GCM in the CMIP5 ensemble (thin grey lines); the mean of all models (thick black line); and the slope of the linear fit to EECRA observations (i.e. derived from data presented in Fig. 2). The maps were created using the NCAR Command Language (Version 6.0.0) [Software] (2011). Boulder, Colorado: UCAR/NCAR/CISL/TDD. http://dx.doi.org/10.5065/D6WD3XH5.

We now study trends derived from the GCMs included in CMIP5 and run under “historical” forcing conditions. The period analyzed is 1971–2005, and the main question is: can CMIP5 historical simulations reproduce observed TCC trends? It should be noted that the reliability of CMIP5 GCMs behavior regarding other spatial and temporal characteristics of TCC has been assessed elsewhere^29^. The top of Fig. 4 shows that the multi-model average (MMA, using 44 models, only 1 run per model), gives an overall decrease in TCC over the Mediterranean. The result shown in the middle panel of Fig. 4 indicates that most models (i.e., more than half) give negative trends (irrespective of the specific value) for all cells in the region. This means that the decreasing trend of TCC is consistently captured by most GCMs. Specifically, the signal is generally more robust in the western Mediterranean Sea, and in the Iberian, Italian, and Balkan peninsulas, and in southern France. It is worth noting that this spatial structure is not totally in agreement with the observed (EECRA) trends, since the pattern of the latter is much more complex as shown in Fig. 1 (top).

In addition, the best estimate of the trend of the mean TCC series of the MMA (only for the EECRA mask cells) is −0.31% decade^−1^ (*p* < 0.01) which is about one half of the observed decrease (−0.63% decade^−1^, *p* < 0.05, as above mentioned) (Fig. 4, bottom). It should be noted, however, that the observations likely reflect a combination of internal variability and the response to external forcing agents, while the variability in the MMA is minimized as the internal variability is uncorrelated across models, so the trend of the MMA may reflect the response to external forcing only. In fact, the apparent discrepancy between the trends (EECRA and MMA) turns into a good agreement if we consider the confidence intervals associated to both of them. Thus, the range associated to the MMA can be estimated by considering the 95% (66%) of the single model trends closest to the median: this gives [−1.0, +0.26] ([−0.60, −0.04]) (all trends in % decade^−1^). It is worth noting that 39 out of the 44 models show a negative trend, indicating great consistency across the different models. The interval associated to the EECRA trend, derived from the linear regression methodology, is [−1.11, −0.12] ([−0.88, −0.39]). So even in the worst case (66% confidence), there is an overlap between the confidence intervals, meaning that it is likely that the actual trend in TCC over the Mediterranean region in the 1971–2005 period is somewhere in the range [−0.60, −0.39]. Finally, interannual variability is fairly consistent among all 44 models, with a mean coefficient of variation, i.e. the ratio of the standard deviation to the mean multiplied by 100, of 3.9% (with a range of [2.1, 5.7] considering the 95% of the single model values) and similar to the coefficient of variation of the mean annual EECRA series (3.4%). Differences between trends from GCMs and trends from EECRA can be better appreciated by looking at the seasonal trends in Figure S2 (in comparison with Fig. 1). Indeed, Figure S2 shows that TCC trends from the MMA are negative for most cells in the domain and for all seasons. Moreover, the lowest simulated trends (trends closer to 0) appear in spring (recall, the season with the largest observed trends, that is, trends with highest negative values), while negative trends also appear in autumn according to the MMA (recall, the only season with clearly positive trends according to observations). So we could say that CMIP5 model results capture the overall TCC decreasing trend, but also that models slightly underestimate its rate of decrease and do not reflect the observed seasonal behavior.

Several authors suggested a possible external radiative forcing in cloud changes during the last decades^40^,^41^. Thus, for example a study for the tropical Indian and Pacific Oceans showed an agreement in the TCC trends from ground-based observations and CMIP5 historical simulations, but, like ours, with smaller magnitudes for the simulations^42^. Recently, large-scale patterns of cloud changes between the 1980 s and the 2000 s have been shown to be in line with CMIP5 simulations with historical forcing^43^. This latter study shows a widespread decrease in TCC in mid-latitudes since the early 1980 s in both observations and simulations, except for example the Mediterranean region where the decrease in the simulations is not observed in the ISCCP and PATMOS-x satellite products. Thus, starting in the 1970 s (as in our study) instead of the 1980 s adds additional evidence of a widespread decrease in mid-latitude regions, highlighting the need of longer time periods to detect statistically significant changes in clouds.

The observed and simulated trends of TCC seem to be in agreement with the poleward expansion of the Hadley circulation cell during recent decades^44^,^45^,^46^,^47^,^48^,^49^, as recently suggested^50^. Specifically, the downward branch of the Hadley circulation can be associated with the edges of the tropical high pressure belt near 30° N and 30° S. Thus, a shift to northern latitudes of the tropical belt edge can lead to substantial changes in the water cycle, including an increase of potential evapotranspiration and decrease in clouds and precipitation in subtropical climate areas such as the Mediterranean region^46^. The widening of the Hadley cell and expansion of the tropical climate is estimated to have occurred at a rate of around 0.5°–1.0° decade^−1^ since the 1970 s^46^, although it is worth noting that this expansion is robustly larger than predicted by the models^49^. Previous studies have suggested that the expansion has been externally forced by tropospheric ozone and black carbon emissions^45^, ozone depletion^51^, greenhouse gases^52^, and warming sea surface temperatures^53^,^54^, although internal low-frequency climate variability may also play a role^46^,^49^. A combination of these factors seems to explain the Hadley cell expansion, but the relative contribution of each forcing is unknown, as well as possible seasonal differences in the rates of the expansion^46^.

This study adds considerable strength to the evidence of widespread decrease in TCC across the Mediterranean region during the last four decades and in particular during the 1970 s–1980 s. This decrease is more evident in winter and especially spring, whereas autumn shows an opposite tendency with a slight and significant increase in TCC during the study period. Equally, there is good agreement between month-to-month and interannual variations of TCC since the mid-1980 s reported by surface observations, satellite datasets, and reanalysis products. Nevertheless, satellite datasets and reanalyses tend to overestimate the decrease in TCC observed at the surface, which highlights the need of an assessment of their temporal homogeneity. Finally, CMIP5 simulations reproduce a decrease in TCC over the Mediterranean region since the 1970 s, in agreement with surface observations as there is an overlap between the trends from the GCMs and from observations, if we consider the respective uncertainties. The trend derived from the multimodel average is slightly lower than that from observations, and climate simulations fail to reproduce the observed seasonal differences in TCC trends. Overall, the results of this study are in line with the observed expansion of the Hadley circulation during the late 20^th^ century. Further research is needed in order to identify the specific mechanisms and forcings responsible for the decreasing trends in TCC over the Mediterranean region, especially regarding their seasonal differences.

## Data and Methods

The main dataset used in this study is built upon human observations of TCC from the surface. Specifically, the Extended Edited Synoptic Cloud Reports Archive (EECRA) is used; this dataset in the last version available provides a collection of individual cloud reports from weather stations over land from 1971 to 2009^32^. The stations used in ECCRA were selected after a screening and quality control procedure^14^,^32^. Here, a TCC grid of 2° × 2.5° (latitude × longitude) has been created in order to minimize biases due to the non-homogeneous spatial distribution of the stations and for a better comparison with the global gridded products and climate simulations. For more details about the gridding method, we refer to ref. 30.

Other global gridded databases have been selected for comparison against the gridded EECRA records. Specifically, we have chosen databases from several satellite projects (ISCCP, CLARA, PATMOS-x), and reanalysis products (MERRA, NCEP-CFSR), which are described in the following paragraphs.

The ISCCP is a project of the World Climate Research Programme (WCRP) that derives cloud and radiative variables from polar and geo-stationary satellites, with the current record extending from 1983 to 2009. For more details, we refer to refs 55 and 56. In this study we use ISCCP TCC monthly mean values provided in the D2 dataset on a grid of 280 × 280 km^2^.

PATMOS-x provides records since 1979 as derived from the National Oceanic and Atmospheric Administration (NOAA) Advanced Very High Resolution Radiometer (AVHRR) sensors^57^,^58^. In this study, we have made use of PATMOS-x TCC in the level 3 data with 1° × 1° of spatial resolution and monthly temporal resolution as provided in the Global Energy and Water Cycle Experiment (GEWEX) Cloud Assessment project (http://climserv.ipsl.polytechnique.fr/gewexca/) from 1982 to 2009^16^.

The CM SAF cLoud, Albedo & Radiation dataset (CLARA) has been developed by the EUMETSAT Satellite Application Facility on Climate Monitoring (CM SAF) project. The grid has a resolution of 0.25° × 0.25° and is available from 1982 to 2009^59^. The cloud and radiation fluxes in CLARA are derived by using the same AVHRR sensors as in the PATMOS-x project. Nevertheless, it is worth noting that cloud variables are produced by means of different algorithms (for more details, see ref. 59).

Regarding reanalysis products, we have used the MERRA and NCEP-CFSR products. In brief, MERRA is a reanalysis developed by NASA for the satellite era that makes use of the Goddard Earth Observing System Data Assimilation System Version 5 (GEOS-5). Its spatial resolution is 0.5° × 0.67° and it provides data since 1979^60^. The NCEP Climate Forecast System Reanalysis (CFSR), which is the most recent reanalysis developed by the NOAA’s NCEP, is a coupled atmosphere-ocean-land surface-sea ice system that provides outputs in a resolution of 0.5° since 1979^61^.

In addition, we have used the simulations of the historical experiment of the Coupled Model Intercomparison Project Phase 5 (CMIP5)^62^. Specifically, we have obtained the monthly TCC values from the first realization of 44 Global Climate Models (GCMs) available from 1850 to 2005. These models are summarized in ref. 30.

In this study, all observational and climate model simulation databases were interpolated to the grid used for EECRA (2° latitude ×2.5° longitude). The interpolation was made using bilinear interpolation by using the function “linint2” implemented in the National Center for Atmosphere Research (NCAR) Command Language (NCL).

It should be noted that not all grid-cells have suitable data from the EECRA dataset. Thus, we have selected only those grid cells that have data available during at least 80% of the study period; these cells will be referred to as the “EECRA mask”. The period 1971–2005 is chosen for the comparison between EECRA observations and GCMs simulations, as 2005 is the last year available for all the CMIP5 historical simulations. Similarly, we have used the time period common to all other datasets (1984–2009) for the comparison of the satellite-derived and reanalysis products against the surface observations.

The trend analyses and the comparisons among datasets were carried out for the entire Mediterranean region, which is defined here as the area between 30–48°N latitude and 10°W–40°E longitude, as well as for each individual cell of the domain. As a first step, time series of annual and seasonal mean values of TCC were derived for each grid point. The seasons are defined as spring (MAM), summer (JJA), autumn (SON), and winter (DJF). The linear trends of the series shown in this paper were calculated by means of least squares linear fitting and where required their significance was assessed by the Mann-Kendall nonparametric test.

## Additional Information

**How to cite this article**: Sanchez-Lorenzo, A. *et al*. Fewer clouds in the Mediterranean: consistency of observations and climate simulations. *Sci. Rep.* **7**, 41475; doi: 10.1038/srep41475 (2017).

**Publisher's note:** Springer Nature remains neutral with regard to jurisdictional claims in published maps and institutional affiliations.

## Supplementary Material

Supplementary Information
