# Supplementary material for: Fewer clouds in the Mediterranean: consistency of observations and climate simulations
Source: Sci Rep. 2017 Feb 2;7:41475. doi: 10.1038/srep41475 (PMC5288658; doi:10.1038/srep41475)
Supplement: Supplementary Information [file srep41475-s1.doc]

**Supplementary Information**

**Fewer clouds in the Mediterranean: consistency of observations and climate simulations**

Arturo Sanchez-Lorenzo^1*^, Aaron Enriquez-Alonso^2^, Josep Calbó^2^, Josep-Abel González^2^, Martin Wild^3^, Doris Folini^3^, Joel R. Norris^4^, Sergio M. Vicente-Serrano^1^

*^1^Instituto Pirenaico de Ecología, Consejo Superior de Investigaciones Científicas (IPE–CSIC), Zaragoza, Spain*

*^2^Department of Physics, University of Girona, Girona, Spain*

*^3^Institute for Atmospheric and Climate Sciences, ETH Zürich, Zürich, Switzerland*

*^4^Scripps Institution of Oceanography, University of California, San Diego, La Jolla, CA, USA*


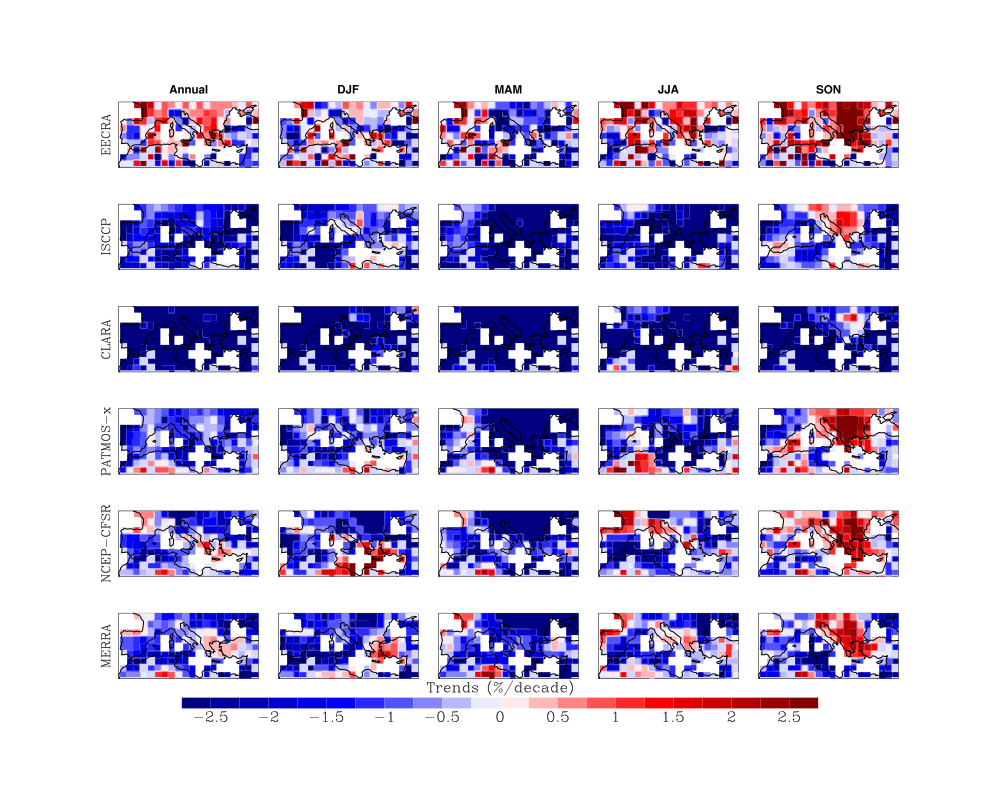
Figure S1. Linear trends of TCC, in the Mediterranean region for the period 1984-2009, derived from ground-based observations (EECRA), satellite projects (ISCCP, CLARA, PATMOS-x), and reanalysis products (NCEP-CFSR, MERRA). It should be noted that only cells considered from the EECRA dataset have been shown for the other databases (despite these databases have a complete spatial cover), in order to facilitate the comparison. Thus, white cells correspond to areas with no data in the EECRA dataset. The maps were created using the NCAR Command Language (Version 6.0.0) [Software]. (2011). Boulder, Colorado: UCAR/NCAR/CISL/TDD. http://dx.doi.org/10.5065/D6WD3XH5.


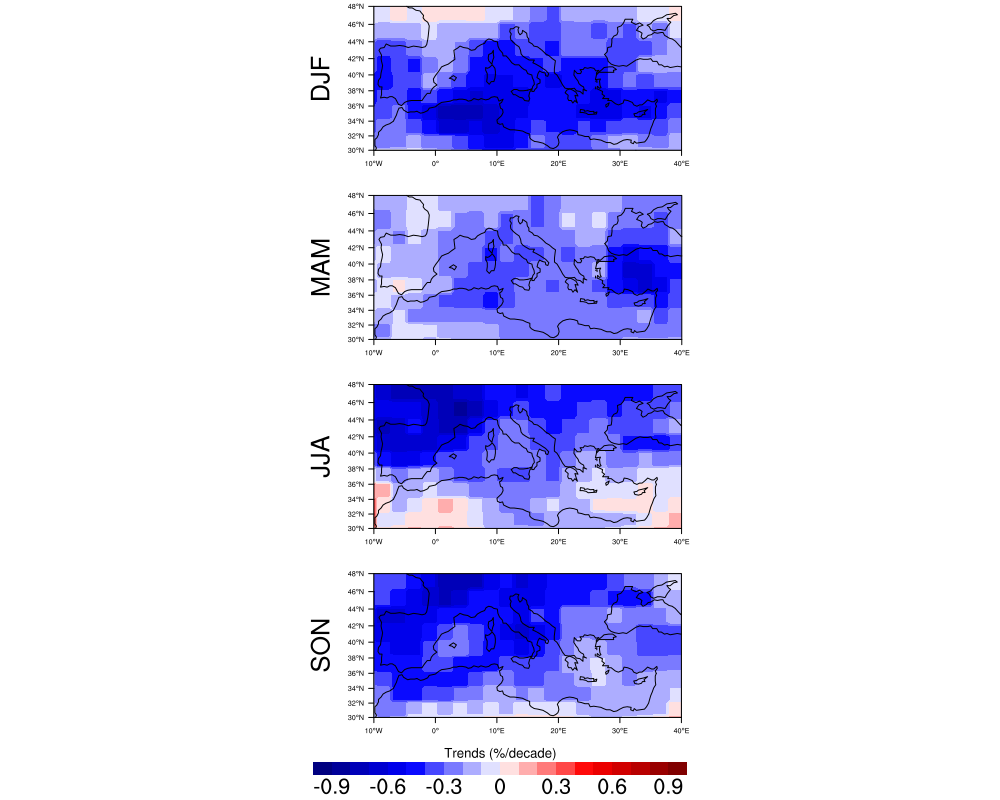

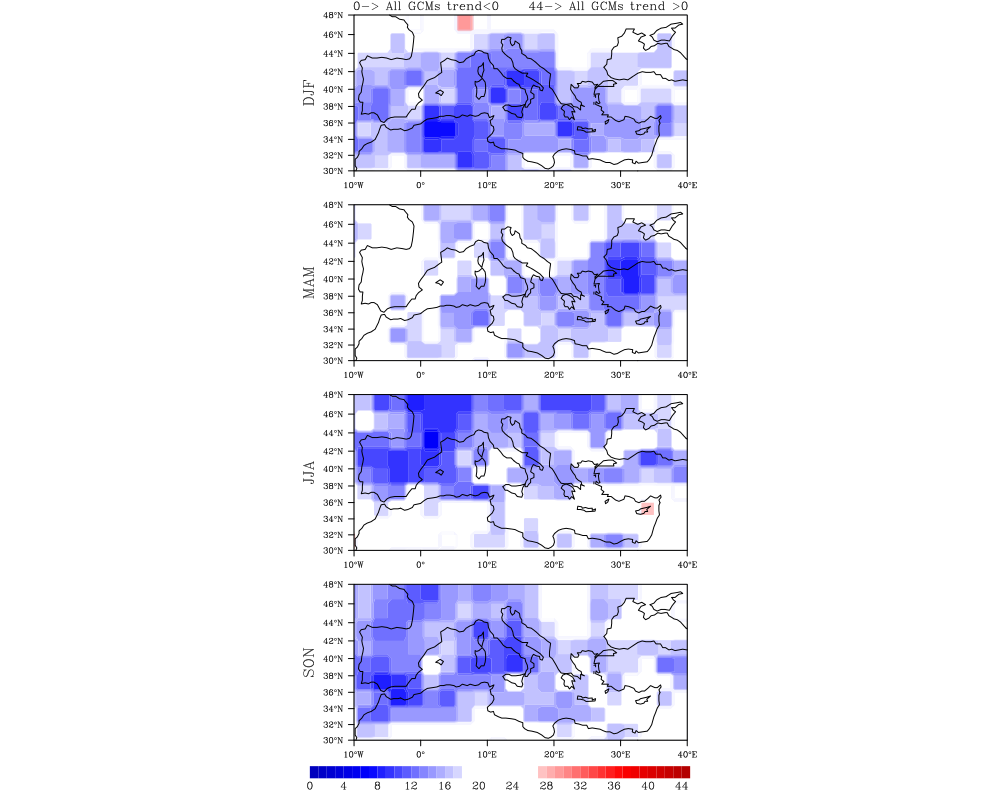


Figure S2. (left) TCC trends (1971-2005) obtained by averaging the trends of the 44 CMIP5 GCMs for each season. (right) Number of GCMs that give a positive TCC trend; therefore, the bluer a cell is tinted, the more robust the negative trend given by the ensemble of the GCMs. The maps were created using the NCAR Command Language (Version 6.0.0) [Software]. (2011). Boulder, Colorado: UCAR/NCAR/CISL/TDD. http://dx.doi.org/10.5065/D6WD3XH5.
